# Imaging of hypoxia in human glioblastoma with dynamic 18F-fluoromisonidazole PET

**DOI:** 10.20517/cdr.2018.21

**Published:** 2019-03-19

**Authors:** Redha-Alla Abdo, Frédéric Lamare, Philippe Fernandez, M’hamed Bentourkia

**Affiliations:** ^1^Department of Nuclear Medicine and Radiobiology, Université de Sherbrooke, Sherbrooke, QC J1H5N4, Canada.; ^2^Service de Médecine Nucléaire, Université de Bordeaux-II, EPHE, Bordeaux 33076, France.

**Keywords:** PET, 18F-fluoromisonidazole, glioblastoma, hypoxia, tumor-to-blood uptake ratio, compartmental modeling

## Abstract

**Aim:** The purpose of this study was to locate the levels of hypoxia in glioblastoma PET images measured with 18F-fluoromisonidazole in human subjects. It is recognized that tumors with hypoxia are resistant to treatment by radiotherapy and chemotherapy.

**Methods:** The images were acquired in dynamic mode for 15 min or 30 min and in static mode for two single scans at 2 h and 3 h to allow the accumulation of the radiotracer in the tumor. The images were analyzed at the voxel basis with compartmental analysis (CA) and with the usual tumor-to-blood uptake ratio (TBR). Kmeans algorithm was applied to cluster the levels of hypoxia in the images.

**Results:** TBR at a threshold of 1.2 at imaging times of 15 min, 2 h and 3 h produced images with different clusters. Also, the comparison of TBR with the distribution volume obtained with CA had a similarity index of 0.61 ± 0.05.

**Conclusion:** We found some differences in defining the hypoxic volume within a tumor using TBR. The compartmental analysis allowed discrimination of the tumor hypoxic sub-volumes which can be useful for a better treatment with radiotherapy.

## Introduction

Glioblastoma multiforme (GBM) is known as the most common tumor in the central nervous system. The current techniques of treatment for GBM include surgery, chemotherapy and radiotherapy. These strategies of treatment have done minimum outcomes in extending the life expectancies of patients diagnosed with GBM. The patients’ median survival is ranging from 1 to 1.5 years, and only 5% of the patients reach five years survival^[1]^.

The difficulty of treating GBM tumors lies in its inherent histological complexity and to various mechanisms leading to treatment resistance. One of these mechanisms is hypoxia, which is defined as low oxygenation levels inside the tumor.

The apparent reason for treatment resistance due to hypoxia in chemotherapy is the tumor’s distance from vasculature which prevents the therapeutic agent from reaching the tumor bed^[2]^. Moreover, the non-proliferation nature of hypoxic tumor can intrinsically immune the tumor to the anti-proliferative anti-cancer drugs^[3]^. The recent studies found that the molecular basis for this resistance belongs to hypoxia inducible factors, which play an essential role in tumorigenesis and angiogenesis^[4]^. In radiotherapy, the effect of treatment resistance due to hypoxia is associated with the oxygen effects on the radio-sensitivity of the tumor^[5]^. For these reasons, there are critical needs for the development of more effective therapeutic approaches.

One of the emerging strategies to overcome hypoxia is to define the hypoxic sub-volumes in the tumor using functional imaging for the goal of radiotherapy dose escalation. This hypoxia dose painting aimed to reach a biological instead of physical conformity for the delivery of radiation to the tumor. The first trial of this technique proposed by Ling *et al*.^[6]^ who applied a non-uniform radiation dose based on tumor biological characteristics.

A variety of PET tracers were used to image hypoxia, such as 18F-fluoromisonidazole (18F-FMISO) and 18F-fluoroazomycin arabinoside^[7]^. 18F-FMISO accumulates in the hypoxic cells for a long time (4 h) *in vivo* and *in vitro*. Furthermore, it requires a high level of hypoxia. However, 18F-FMISO only accumulates in viable cells with the presence of hypoxia, which can be advantageous for *in vivo* imaging^[8]^.

For hypoxia measurement in PET images, tumor-to-blood uptake ratio (TBR) is the commonly used parameter for single time frame images. It is described as the ratio of the radiotracer concentration in the tumor cells to its concentration in blood^[9]^. However, TBR does not give any information about the kinetics or changes of the tumor hypoxia with time. Compartmental analysis (CA) with kinetic modeling is the mostly used approach to analyze the dynamic PET data^[10]^. This method is based on the design of a mathematical model which best describes the physiological processes in which the tracer is involved^[11]^.

The objectives of this study were to demonstrate the variation of TBR with PET acquisition time, and to decompose the dynamic 18F-FMISO PET images into clusters describing hypoxia sub-volumes. This decomposition would allow the oncologists to optimize the treatment dose.

## Methods

### Subjects’ data

Nine human subjects with GBM tumors were imaged with PET/CT and 18F-FMISO radiotracer. 18F-FMISO was prepared using the method as described in^[12]^. The PET image acquisition started with the injection of 3.7 MBq/kg of 18F-FMISO in a dynamic acquisition of 15 min or 30 min using the PET scanner Discovery RX General Electric Medical System. Then the subjects left the scanner. Two additional static images were obtained for all subjects at 2 h and 3 h post-injection (pi). The images were reconstructed in 12 frames of 10 s, 8 frames of 15 s, 11 frames of 60 s and 600 s for the two static images. The dynamic PET images from 0 to 15 min or 30 min were coregistered with those at 2 h and 3 h static images based on the coregistration of their respective CT images.

### Data analyses

#### TBR analyses

TBR images were estimated by dividing the PET images by the blood value obtained from the input function (IF) at the corresponding frame time. IF was derived for each subject from the carotid artery in PET images by drawing a region of interest (ROI) on the sagittal view to encompass a large area of the carotid. Hypoxia images were then obtained by thresholding TBR images acquired at 15 min, 2 h and at 3 h, at a level of 1.2 which is the commonly used level in the literature^[9]^.

#### Compartmental analysis

We used the two-tissue compartment model for kinetic modeling of the dynamic images^[13,14]^ at the voxel basis in order to obtain parametric images. This model is based on the physiological properties of 18F-FMSIO representing the exchange of the radiotracer with blood stream in the perfused compartment of the tumor and its accumulation in the hypoxic compartment.

The time-activity curve (TAC) of the radiotracer concentration in blood was assessed by drawing an ROI on the carotid artery from summed sagittal images on the first 4 min where the images show only blood. The large sagittal images of the carotids allowed to minimize the effect of partial volume. The ROIs on the tumors were fitted with the two-tissue compartment model^[13,14]^ using the Levenberg-Marquardt algorithm. The adjustment of the tumor TAC in each pixel returned the four rate constants *K*_1_ - *k*_4_ and blood volume.

The distribution volume was estimated as *K*_1_/(*k*_2_ + *k*_3_)^[13]^, where *K*_1_ is the perfusion rate constant, *k*_2_ is the brain to blood transport rate constant (reverse capillary membrane transport), and *k*_3_ is the 18F-FMISO retention rate constant in the tumor. Distribution volume is a more sensitive parameter for hypoxia determination as it depends on the perfusion *K*_1_ and on the extraction and elimination rate constants *k*_3_ and *k*_2_ respectively^[13,14]^, instead of relying on the perfusion rate constant *K*_1_ as reported in^[15]^ where the authors compared *K*_1_ to the perfusion measured with perfusion CT imaging in lung cancer.

#### Statistical analyses

CA modeling and statistical analyses were implemented in MATLAB (R2015a, The Math Works Inc., Natick, MA, USA). The CA distribution volume was analyzed using Kmeans and elbow method^[16]^. The comparison between TBR and CA images was assessed using the similarity index (SI)^[17]^:


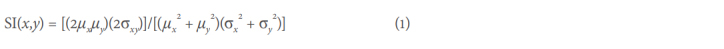


Where *μ*_*x*_, *μ*_*y*_, σ_*x*_, σ_*y*_ and σ_*xy*_ are the local means, standard deviations, and cross-covariance for images *x* and *y*. The SI value of 1 indicates a complete agreement while 0 indicates a disagreement between the two images.

The elbow method is utilized to select the optimal number of clusters in a dataset as returned by Kmeans. For each number of clusters *k*, say from 1 to 10, the sum of the squared errors are calculated and plotted as a function of the values of *k*, which can be seen to sharply drop at a certain value of *k*. This drop indicates the maximum of clusters needed^[18]^.

## Results

**Figure 1:**
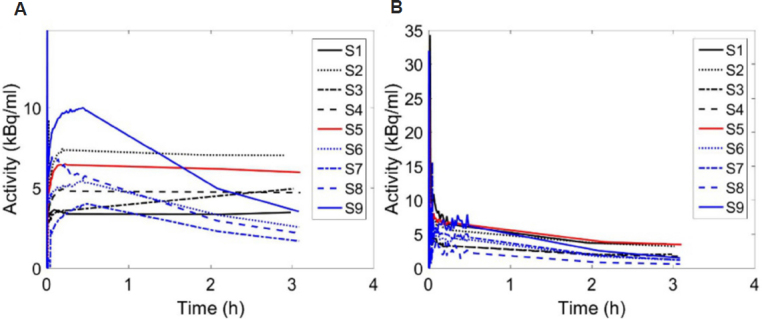
A: Tumor time activity curves in the nine subjects; B: their corresponding input functions

Tumor TACs defined by drawing ROIs on the tumor images from the nine subjects are shown in Figure 1A. The uptake of 18F-FMISO presented different behavior as a function of time among subjects. The increasing TACs were related to hypoxic tumors accumulating 18F-FMISO, those having a plateau represented stable and uniform hypoxia including necrotic sub-volumes, and those with decreasing TACs were related to perfused tumors with less hypoxia. Figure 1B shows the IFs in the respective subjects as for the TACs.

**Figure 2:**
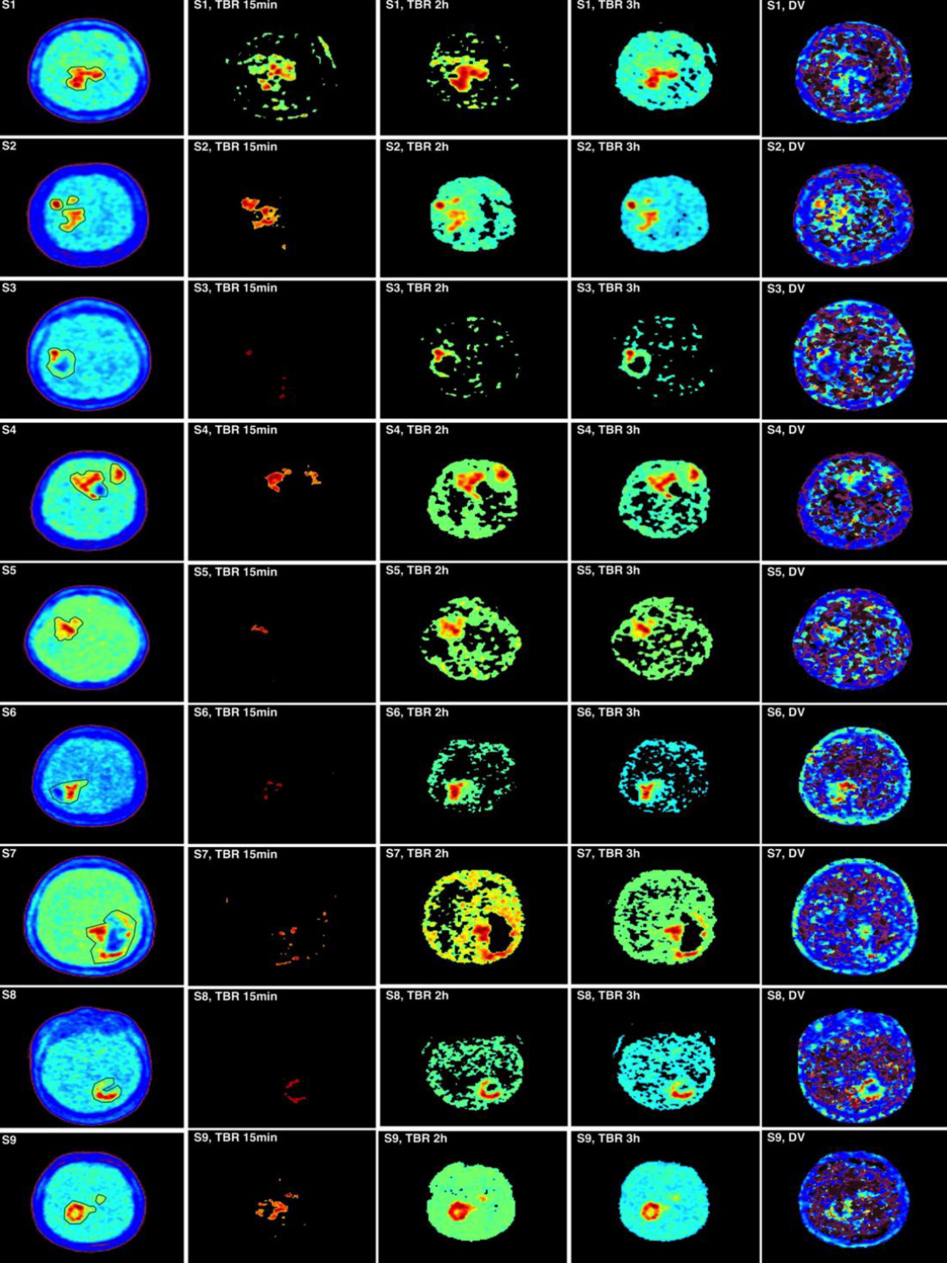
Comparison of tumor-to-blood uptake ratio (TBR) at 15 min, 2 h and 3 h with compartmental analysis (CA) images. The first column shows the measured PET images at 3 h pi with region of interests around the tumor. The three-centered columns are for the thresholded TBR images at a threshold of 1.2. The fifth column is the corresponding CA distribution volume

Figure 2 first column depicts images of the nine subjects taken from the image frames at 3 h, where the tumor ROIs were also drawn. ROIs in some subjects are shown to encompass deeply contrasted volumes (Figure 2, first column: S3, S4, S6 and S7) which we assumed to be necrotic sub-volumes. In all images, at 3 h, the tumor appeared well contrasted from the rest of the brain which was perfused, indicating that the exchange of 18F-FMISO between the tumor and blood is slow in comparison to the exchange between normal tissue and blood (Figure 2, first column).

Figure 2 shows also TBR for 15 min, 2 h and 3 h pi after 1.2 thresholding. TBR translated the contrast between the tumor and the blood, and consequently between hypoxic and perfused tissues. Depending on the time of the PET measurement, the ratio of tissue to blood radiotracer concentration can drastically vary. At 15 min, the radiotracer concentration in blood is high while the uptake in the tumor has not been optimally achieved (see Figure 1A for tissue and Figure 1B for blood), and consequently TBR values partly isolate tumor hypoxia as shown in Figure 2 second column. For this reason, it has been proposed to delay the PET imaging at 2 h pi or later in order to allow more uptake of the radiotracer in the tumor and concurrently its washout from blood. Still TBR at 2 h and 3 h shows differences in tumor hypoxia definition (Figure 2, columns 3 and 4). Therefore, the time for a single PET scan to isolate hypoxia remains questionable. The large perfused regions in the images defined with TBR can also be explained by the underestimation of the tracer activity in blood as determined from the carotid artery, and this might be partly due to the partial volume effect.

The TBR images at 15 min, 2 h and 3 h highlighted the difference in tumor volume determination which was varying with time. Such assumptions can lead to different results depending on the protocol for 18F-FMISO imaging. The last column in Figure 2 represents the CA distribution volume for each subject. These images can help in understanding the kinetics of 18F-FMISO with time. The similarity test between TBR images at 3 h and the corresponding CA distribution volume images was mean (SI) = 0.61 ± 0.05.

**Figure 3:**
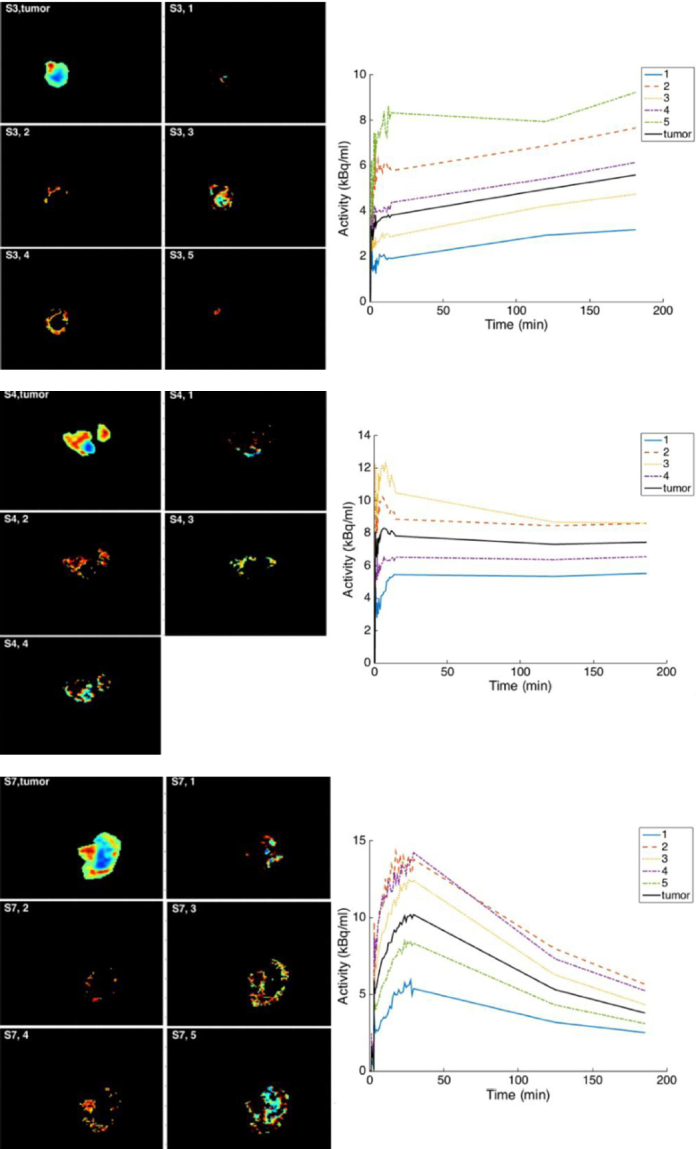
The separation of compartmental analysis (CA) distribution volume based on Kmeans clustering and elbow method. From upper to bottom: S3, S4 and S7. The displayed image of the whole tumor is from the measured image. For each cluster determined on CA distribution volume, its time-activity curve was extracted from the measured images. In these subjects, the accumulation of 18F-fluoromisonidazole is seen to respectively increase, stabilize and decrease

The clustering to discriminate the levels of hypoxia from the distribution volume images are shown in Figure 3 for three subjects. Based on the determination of the clusters, the TACs were produced to show the time course for each cluster [Figure 3]. The three subjects’ data in Figure 3 were selected to show the accumulation with time of 18F-FMISO as increasing, stabilizing and decreasing. Again, CA images were shown to provide different structures of the tumor depending on its uptake of 18F-FMISO. The clusters presented different time course shapes and sub-volumes within the tumor in each subject and these might have reflected the levels of hypoxia.

**Figure 4:**
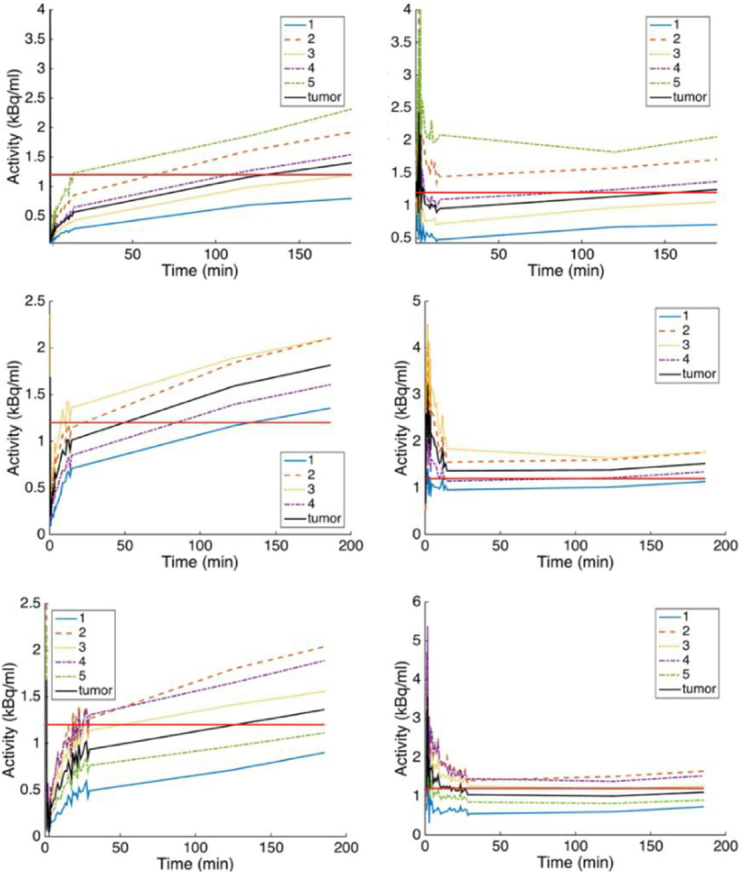
The time-activity curves (TACs) of the distribution volume clusters in Figure 3 were normalized here with IF (left column) and with TAC obtained from a contralateral normal tissue. The straight line represents the threshold of 1.2 above which the cluster was considered as hypoxic

Figure 4 was obtained from the cluster TACs in Figure 3 divided at time points by IF (Figure 4 left column) and by a contralateral normal tissue TAC (Figure 4 right column). In these plots, a horizontal straight line has been added at the level 1.2 to indicate the threshold used in TBR to separate hypoxic from non-hypoxic clusters. Clustering hypoxia reflects more its heterogeneity and possibly provides more accurate treatments.

**Figure 5:**
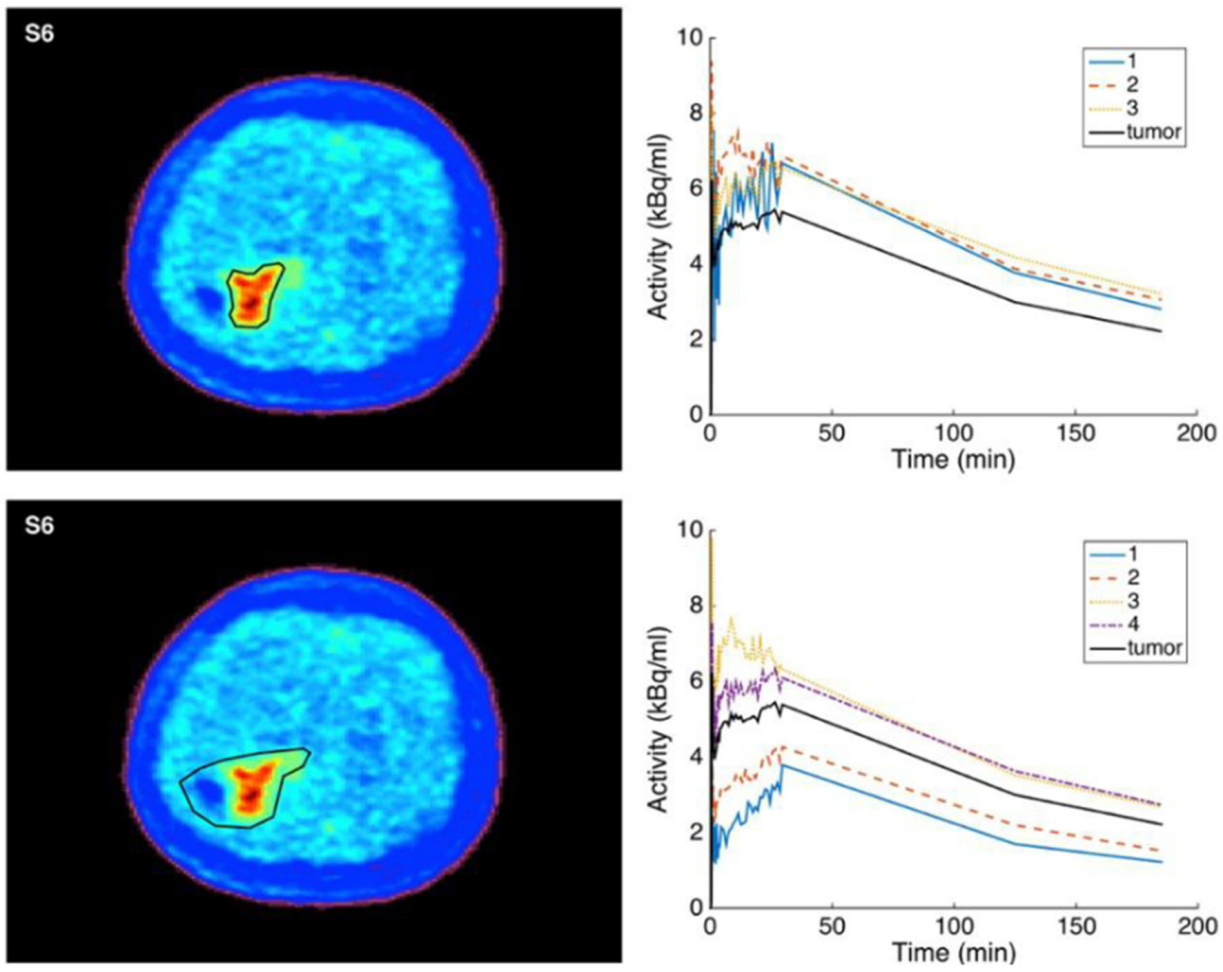
The effect of clustering when including or excluding tumor depressed regions (after surgery without presence of inflammation or in presence of necrosis)

Figure 5 depicts the behavior of the cluster TACs in the same image slice by including or excluding the necrotic region in the tumor. A thresholding or a clustering can resolve the depressed regions in the tumor after application of CA.

## Discussion

There are many major challenges in defining the hypoxic region with 18F-FMISO PET images. For example, the accumulated activity in the voxel depends on the volume of hypoxia in the tumor. Furthermore, the transport properties and acquisition time affect the uptake of 18F-FMISO. Defining the exact volume of hypoxia within GBM tumors can help to overcome the treatment resistance in chemo-radiotherapy. Most PET imaging in radiotherapy used single static scan at 2 h to 4 h pi to allow for 18F-FMISO accumulation in the target cells.

The current assessment of hypoxia using TBR or standard uptake value shows the uptake at a specific time point. Therefore, their results would change by different time measurements^[9]^. This observation is clearly indicated in Figures 1 and 2 where the tumor hypoxic volumes were different between the 15 min, 2 h and 3 h. By using TBR, the boost dose of radiation treatment to the hypoxic region would depend on the scanning time of 18F-FMISO. In addition to scanning time, the threshold value of TBR has not yet been well established. We used the threshold value of 1.2 in this study, while other studies suggesting the value of 1.4. In the literature, many threshold values have been used for delineating the hypoxic volume other than 1.2 or 1.4. In addition, the uncertainties on the 18F-FMISO activity in blood have a reasonable impact on hypoxia determination.

By using CA technique, it was possible to differentiate the voxels inside tumor hypoxic volume into different levels of hypoxia. These differences resulted from the fact that TBR would use only a single time data, while CA evaluates the data using the whole dynamic images. The patterns of the cluster TACs showed different behavior other than the previous shape of the whole tumor (Figure 3 *vs*. Figure 1). Thorwarth *et al*.^[19]^ had described four examples of TACs with 18F-FMISO. These were the well-perfused region TAC, diffusion-limited hypoxia, diffusion-limited with structural hypoxia and high hypoxic/necrotic area.

About hypoxia sub-volumes and levels, most of authors defined two tumor sub-volumes as volumes with signal intensity below or above a given threshold usually taken as TBR = 1.2. Because hypoxia is spatially heterogeneous, the thresholding normally generates clusters of voxels with TBR > 1.2. The same pattern is obtained when applying compartmental modeling^[19]^. Moreover, a single voxel can be made of perfused and hypoxic sub-volumes^[19]^. Since clusters of hypoxic sub-volumes can be found at different locations in the tumor, these clusters can have in average different signals (or Bq/mL) which can be referred to as hypoxia levels. Dirix *et al*.^[20]^ and Zips *et al*.^[21]^ reported the correlation of radiation treatment with hypoxia magnitude as assessed with T/Bmax^[20]^ and with TBR = 1.4, 1.6, 1.8 and 2^[21]^.

In conclusions, we showed in this work some differences in defining the hypoxic volumes within a tumor using TBR with the single static imaging protocol, while the compartmental analysis allowed to determine the levels of hypoxia within the tumor sub-volumes. Because GBM behaves differently from one subject to another, it is suggested to apply the appropriate analysis approach instead of using a common method like TBR. The 30 min dynamic PET data could be appropriate and sufficient to isolate hypoxia levels. Although this approach seems to have potential, more validations are required for its appropriate application. This might possibly be achieved in a randomized study.

## Declarations

### Data source and availability

Corresponding author may be contacted for any data inquiries.

### Copyright

© The Author(s) 2019.
